# High-saturated-fat diet drives female-biased neurodegeneration in *Drosophila* via oxidative stress and impaired autophagic flux

**DOI:** 10.3389/fnagi.2026.1759336

**Published:** 2026-06-15

**Authors:** A. M. Taylor, K. L. Matzeller, E. M. McNeill

**Affiliations:** 1 Food Science and Human Nutrition, Iowa State University, Ames, IA, United States; 2 Graduate Program in Neuroscience and Interdepartmental Graduate Program in Nutritional Sciences, Iowa State University, Ames, IA, United States

**Keywords:** autophagy, *Drosophila melanogaster*, high fat diet (HFD), inflammation, neurodegeneration, oxidative stress, saturated fat

## Abstract

**Objective:**

Dietary saturated fat intake has been increasingly linked to dementia risk, yet the mechanisms through which high-fat diets (HFDs) compromise neuronal integrity remain poorly defined. The model organism *Drosophila melanogaster* may serve as an ideal model to understand these mechanisms across aging due to its genetic tractability and short lifespan.

**Methods:**

Flies were fed diets containing 30% (w/v) fat from 2 oils with varying saturated-fat content and were assessed for lifespan and climbing ability. Immunohistochemistry and confocal imaging were used to evaluate brain tissue integrity, quantify vacuoles, and assess oxidative stress and activation of an inflammatory pathway. Genetically encoded and UAS-driven fluorescent reporters, along with confocal imaging, were used to assess autophagy and autophagic flux.

**Results:**

Both HFDs shortened lifespan but only the highest saturated-fat source (coconut oil) uniquely produced a female-specific decline in climbing ability and significant neurodegeneration, compared to lard and control diets. Mechanistic evaluation revealed no activation of the NF-κB homolog *relish* in response to a coconut oil HFD however chronic HFD feeding led to elevated neuronal reactive oxygen species and early impairment of autophagic flux, preceding autophagosome accumulation during aging.

**Conclusion:**

These findings identify oxidative stress and disrupted autophagy as key mediators of saturated-fat-induced neuronal decline and highlight a sex-specific vulnerability to dietary fat composition. This work establishes *Drosophila* as a powerful model for dissecting nutritional drivers of neural aging and suggests that metabolic stress pathways represent critical early targets in diet-associated neurodegeneration.

## 1 Introduction

Dementia affects more than 55 million people worldwide and is characterized by progressive cognitive decline and neuronal tissue loss (Dementia, n.d.). Both modifiable lifestyle factors, such as diet, and non-modifiable factors, including genetics, contribute to disease risk (Nordestgaard et al., 2022). Animal studies have begun to reveal key cellular mechanisms underlying neurodegeneration. For example, evidence supports increased oxidative stress and alterations in cellular processes in the brains of mouse models of neurodegenerative disease, including inflammation, autophagy, and metabolism (Wareham et al., 2022; Esmaeili et al., 2022; Ribeiro et al., 2013; Bianchi et al., 2021). However, despite this research progress, additional work is needed, particularly regarding how diet may influence these mechanisms and contribute to neurodegeneration. In recent years, *Drosophila melanogaster* has emerged as an ideal model organism for studying the interplay between diet and neurodegeneration, thanks to its short lifespan, rich array of genetic manipulation tools, and high levels of genetic conservation with mammals; yet this model is relatively underutilized in nutritional neuroscience research (Bolus et al., 2020; Cormier et al., 2021; Nitta and Sugie, 2022).

Relatively few studies have examined the effects of a HFD in *Drosophila* compared to mammalian models, but some similarities have been observed. Studies in *Drosophila* show a diet high in fat induces detrimental health effects, including decreased lifespan, impaired reproduction in females, elevated oxidative stress, and altered expression of genes associated with both cellular stress (e.g., heat shock proteins) and metabolic pathways, including glycolysis and lipolysis (Cormier et al., 2021; Liao et al., 2021; Trindade de Paula et al., 2016; Stobdan et al., 2019; Eickelberg et al., 2022; Rivera et al., 2019). Regarding the central nervous system (CNS), evidence indicates that a HFD results in cognitive deficits, a consequence consistent with findings in mammalian models and humans consuming dietary fat (Rivera et al., 2019; Greenwood and Winocur, 2005; Sharma, 2021; Tan and Norhaizan, 2019; Yi et al., 2024). Additionally, a HFD accelerates behavioral aging, leading to an earlier onset of behavioral deficits associated with aging.(Stobdan et al., 2019) Despite these studies, the direct impact of dietary fat on neurodegeneration in the fly CNS remains insufficiently defined.

*Drosophila* is a tractable model for neurodegeneration research, and many mutant lines exist that induce protein aggregates commonly found in such pathologies, leading to tissue loss throughout the brain (Bolus et al., 2020; Nitta and Sugie, 2022; Sakakibara et al., 2023). Additionally, there are many well-established assays to investigate neuronal health at behavioral, biochemical, and morphological levels. As aging is one of the most influential risk factors for neurodegeneration, their short lifespan of 60–80 days allows for observation of the effect of lifelong dietary patterns rather than short-term exposure preceding a given measurement (Staats et al., 2018; Lüersen et al., 2019). These factors, along with the considerable conservation of cellular mechanisms underlying both neurodegeneration and metabolism, position *Drosophila* as a promising model organism for studying mechanisms relevant to human neurodegeneration.

An overload of oxidative stress is detrimental to neuronal homeostasis and can lead to neurodegeneration (Kim et al., 2015). Metabolic processes, particularly those involved in lipid metabolism, produce free radicals, also known as reactive oxygen species (ROS). These oxidative molecules can damage DNA, cell membranes, and mitochondria, and can initiate an inflammatory response but are typically neutralized by endogenous antioxidants. However, when ROS levels exceed the antioxidant defense capacity, cells enter a state of oxidative stress that can trigger apoptosis (Simpson and Oliver, 2020). The brain is particularly susceptible to damage from oxidative stress as it is metabolically active and has a limited antioxidant defense capacity. Studies support that a diet high in saturated fat leads to increased oxidative stress both throughout the body and in the brain, leading to cognitive deficits in mammalian models (Esmaeili et al., 2022; Tan and Norhaizan, 2019). Although there is evidence that a HFD increases whole body ROS but its direct effect within the fly brain has not been determined yet (Trindade de Paula et al., 2016).

Chronic activation of inflammatory pathways within the CNS, known as neuroinflammation, have been tied to the negative effects of aging and implicated in many neurodegenerative diseases (Pasqualetti et al., 2015). There is also a growing body of evidence suggesting that neuroinflammation occurs with the consumption of a HFD (Cavaliere et al., 2019; Fan et al., 2021; Kip and Parr-Brownlie, 2023). A neuroinflammatory response is typically initiated in the presence of an insult such as an injury, ROS, or infiltration of peripheral cytokines through the blood–brain barrier. These insults induce genetic programs that alter the expression of genes and downstream proteins that propagate the inflammatory response. One of the most well-studied inflammatory pathways in neurodegeneration involves the transcription factor nuclear factor kappa beta (NF-κB). This pathway is chronically activated by a HFD in various tissues like adipose and liver. Given the brain’s protected status via the blood–brain barrier, it is uncertain whether NF-κB is activated within the CNS. Recent studies investigating the influence of a HFD on brains of mice have shown increases in neuronal cytokines, such as TNF-α and IL-1β, which are expressed via NF-κB activation, suggesting that it plays a role in the neuronal consequences of a HFD (Santos et al., 2022; Masetto Antunes et al., 2022).

Autophagy is the cell’s natural process that breaks down its components and is vital for cellular homeostasis. It allows nutrient and organelle recycling, stress mitigation, and clearance of cellular debris. It is a multistep process involving engulfment of cargo within a double membrane vesicle known as an autophagosome, fusion with an acidic lysosome, and then degradation of contents and release of amino acids. There is a high degree of genetic regulation at each step of this pathway, and dysregulation at any stage is associated with numerous diseases, including multiple neurodegenerative diseases. Autophagy is closely tied to metabolism, as starvation is a strong activator that enables the cell to mobilize stored nutrients in times of scarcity (Corti et al., 2020). In the context of a HFD, numerous tissues have shown decreases in autophagic activity or disruption of specific steps of the pathway (Khandia et al., 2019). Mammalian studies show that a HFD represses the autophagy-activating AMPK/MTOR pathway, thereby decreasing overall autophagy (Yi et al., 2024; Hou et al., 2022). Others show a disruption of autophagic flux, meaning autophagy initiates normally but does not effectively complete degradation of cargo (Hou et al., 2022). Similar to mammals, disruptions in autophagy and autophagic flux in various peripheral tissues are linked to a HFD in *Drosophila* (Lőrincz et al., 2017; Huang et al., 2020). Additionally, genetically induced deficits in autophagy or autophagic flux, particularly within the cells primarily responsible for CNS lipid metabolism, known as glia, can lead to neurodegeneration in *Drosophila* (Santarelli et al., 2023; Demir and Kacew, 2023).

The aim of this study was to characterize how a high-saturated-fat diet affects the *Drosophila* CNS and to evaluate key neurodegeneration-associated pathways, including autophagy, inflammation, and oxidative stress. Two commonly used dietary fat sources, coconut oil and lard, were selected because they differ substantially in saturated fat content (92% vs. 41% of total fat, respectively) (Kenneth, 2024), enabling us to investigate whether fat composition versus total fat influences neuronal outcomes. We assessed multiple indicators of organismal and neuronal health, including lifespan, negative geotaxis, and CNS tissue morphology. In addition, we examined oxidative stress levels, NF-κB signaling, and autophagic flux to identify cellular mechanisms linking dietary fat to neurodegeneration. As autophagy, oxidative stress and neuroinflammation are strongly associated with neuronal homeostasis and are highly implicated in neurodegenerative diseases, these pathways were prioritized as logical candidates connecting dietary fat to neuronal integrity.

## 2 Methods

### 2.1 Fly husbandry and diet formulation

Flies were cultured at 25 °C and under 60% humidity with a 12:12 h light–dark cycle. The isogenic *w^1118^* were used throughout the study unless otherwise noted. Standard diet was formulated according to Bloomington *Drosophila* Stock Center (Indiana University) and contains the following ingredients: 803 mL H_2_O, 14.6 g yeast, 81.2 g cornmeal, 24.5 g sucrose, 48.8 g dextrose, 9.5 mL 45% propionic acid, 11 mL 17% tegosept and 7.5 g Agar. Agar and half the water were combined with water and heated to 80 °C via a double boiler, at which point the remaining water and dry ingredients were added and mixed well until homogenous. Food was cooled to 65 °C at which point the preservatives propionic acid and tegosept were mixed in, and 2–4 mls were piped into standard plastic *Drosophila* vials. This standard diet was used as the control diet for all experimental assays. The high-fat diet (HFD) was made in the same manner using the following ingredients: 762 mL H_2_O, 14.6 g yeast, 81.2 g cornmeal, 23.4 g sucrose, 46.6 g dextrose, 9 mL 45%propionic acid, 10.5 mL 17% tegosept and 8 g Agar, 77.8 g of either extra virgin, unrefined coconut oil or lard to achieve 30% fat (w/v). Fats were gently heated until they were in liquid form and incorporated into the food mixture at the same step as the yeast, sugars, and cornmeal. Flies were collected within 24 h of eclosion and maintained on the standard diet for 7 days to allow for mating and neurodevelopment to be complete. Male and female flies were then anesthetized via CO_2_, sorted and transferred to either a HFD or control diet-containing vial in groups of no more than 10 flies per vial until the experimental assay was carried out. All vials were maintained in a horizontal position and a small strip of Kimwipe was provided as a dry, clean space for flies to rest.

### 2.2 Lifespan

Seven-day-old sex-matched flies were allocated to the control or one of the HFDs in groups of 10 flies per vial. Flies were maintained under standard conditions described above, and the number of dead and alive flies were noted daily until there were no remaining flies left on the HFD condition. Food medium was renewed two times a week at which point dead flies were removed from the vial. Biweekly transfers to fresh food did not use any anesthetization but rather the standard “flipping” method: tapping all flies to the bottom of their vial, then quickly flipping the old vial upside down on top of the new vial so both open ends aligned and gently tapping so all flies fell into the new vial.

### 2.3 Negative geotaxis

Flies were collected, aged and sorted into dietary groups as described above and further aged for an additional 24 h (1 day time point) or 13 days (13 day time point). 8–10 flies were transferred directly from food vial into an empty standard vial (climbing vial) with markings every 0.05 cm along the side using the standard flipping method described above. After a 20 min acclimation period the induced movement (negative geotaxis) of the flies was initiated by applying 3 sharp taps of the vial on the benchtop surface, causing flies to drop to the bottom of the climbing vial. A photo was taken 3 s after the third tap using a stabilized iPad with a photo timer. This was repeated 6 times for all samples with no less than 2 min of rest provided between trials. The height that each fly climbed within that vial was recorded from the image taken and was averaged across the number of flies for that trial. The average height climbed for the sample was determined by averaging the 6 trial heights. Independent assays were performed for each time point.

### 2.4 Neurodegeneration

Vacuole analysis was conducted according to Behnke et al. (2021) In brief, flies were anesthetized using CO2 after rearing according to standard conditions and methods described above, 13 days after being placed on either a control or a HFD. They were then submerged in 4% paraformaldehyde solution in 1.5 mL Eppendorf tubes overnight at 4 °C. Samples were then washed 3X for 20 min each with 0.5% tween in phosphate-buffered saline (PBST) before brains were dissected in ice-cold 0.008% PBST under a dissecting microscope. Samples were then incubated in a 1:100 Alexa Fluor™ 488 Phalloidin (Thermo Fisher Scientific, AB_2315147) and 1:1000 DAPI (Invitrogen, AB_2629482) cocktail in 0.5% PBST on a light-protected shaker for 24–36 h at 4 °C. Samples were then washed 3X for 15 min with 0.5% PBST and once for 30 min in PBS before being mounted in Slowfade Antigold^tm^ (Thermo Fisher Scientific, S36937) and sealed under a coverslip (#1.5 thickness) with clear nail polish. Confocal imaging was conducted using an Olympus Fluoview FV3000 scanning confocal scope, 60x objective, and at wavelengths of 488 and 405 nm. A z-stack was taken for the central lobe and the number of vacuoles ≥ 1 um were counted and averaged for each diet condition using FIJI software. Treatment groups were blinded for analysis.

### 2.5 Oxidative stress

Total cytosolic ROS was detected and measured according to Biosa et al. (2018) In short, fly brains were dissected in ice-cold PBS and then incubated in 30 μM Dichlorofluorescein (H2DCFDA; Sigma, D399) for 10 min before being washed 3X with PBS, sealed with a coverslip (#1.5 thickness) and clear nail polish, and imaged immediately using a ZEISS fluorescent microscope with a 40x objective. Fluorescent intensity was measured by manually tracing the region of interest (ROI) of the entire brain using the freehand selection tool in Fiji and then measuring the intensity density within that ROI. This value was normalized to the average intensity of three background (non-brain) ROIs within the same image. Background ROIs were generated using the oval selection tool in Fiji and applied consistently across all images to ensure uniform normalization. Control samples were dissected alongside experimental HFD samples and comparisons were made only between samples dissected on the same day to minimize variability commonly associated with H2DCFDA. ROS measurements were obtained at two independent time points; the first being after a single day of exposure to either coconut oil or lard HFD, and the second after 13 days of exposure to these dietary treatments.

### 2.6 Relish expression and activation

Relish expression and activation were measured using immunohistochemistry (IHC) on whole mount brain samples. Animals were reared and dissected as described in the neurodegeneration assay. Samples were first incubated in 1:50 mouse anti-Relish 110 (Developmental studies Hybridoma Bank AB_1553772) and 1:300 rabbit anti-Relish 68 (RayBiotech NP_477094) in PBST at 4 °C overnight then in 1:400488 anti mouse (Invitrogen, AB_2534069) and 1:400568 anti rabbit (Molecular Probes, AB_143157) and 1:1000 DAPI (Invitrogen, AB_2629482). Samples were then washed 3X with PBS before being mounted on slides in SlowFade Antigold^tm^ (Thermo Fisher Scientific, S36937) and sealed with a coverslip (#1.5 thickness) and clear nail polish. Imaging was conducted using a confocal microscope, with a 60x objective and wavelengths of 405, 594, and 488 nm. Analysis consisted of measuring fluorescence in Fiji for all 3 wavelengths within 5 ROIs per treatment-blinded image. Two images were taken per animal, translating to 10 total ROIs per sample. ROI’s were generated using the oval selection tool to outline a singular cell in the DAPI channel.

### 2.7 Autophagy and autophagic flux

Autophagy and autophagic flux were measured using the UAS-gal4 driven ATG8a with a tandem GFP-mCherry reporter obtained from Bloomington stock center (BDSC_37749). This line encodes for the ATG8a gene, a vesicle membrane protein important for autophagic initiation and lysosomal fusion, homologous to mammalian LC3, with the two fluorescent proteins GFP & mCherry. Males from this line were crossed with the glia driver Repo-gal4 virgin females. The F1 offspring from this cross were collected within 24 h of eclosion and maintained on standard conditions as described above for 7 days before being sorted into males and females and placed on either coconut oil HFD or control diet. The same two independent time points used throughout this study were used in this assay (i.e., 1 and 13 days of exposure to a HFD). After the indicated days of exposure, fly brains were dissected and fixed with 4% PFA as described above and immediately mounted in Slowfade Antigold ™ (Thermo Fisher Scientific, S36937) and sealed with a coverslip (#1.5 thickness) and clear nail polish. Z-stacks of the central lobes for each dissected sample were obtained via confocal microscopy using a 60x lens and 488 and 594 nm wavelengths. Analysis was conducted with the Fiji imaging software and h-maxima interactive watershed plugin as described in Brazill et al. (2018) using 3 biological replicates, each calculated using the average of 3 ROIs per replicate. ROIs were generated using the oval selection tool and applied across all sample images. Treatment groups were blinded during analysis.

### 2.8 Statistical analysis

All data are represented as mean ± SEM. Lifespan analysis was conducted using a Mantel-Cox log rank test. A two-way ANOVA was used to assess differences in both the climbing and neurodegeneration assays to determine effects of diet and/or sex, followed by a one-way ANOVA and Tukey’s *post hoc* analysis when appropriate. A student’s T-test with Bonferroni correction was used to determine statistical differences between coconut oil HFD and control diet, or lard HFD and control diet in ROS measurements. A Student’s T-test was used to test for differences between coconut HFD and control diet in Relish analysis, as well as for autophagic vesicle analysis, and autophagic flux analysis. Differences were considered significant at *p* ≤ 0.05 while trends were reported at *p* ≤ 0.1.

## 3 Results

### 3.1 Sex and dietary fat source differentially affect lifespan under a high-fat diet

**Figure 1:**
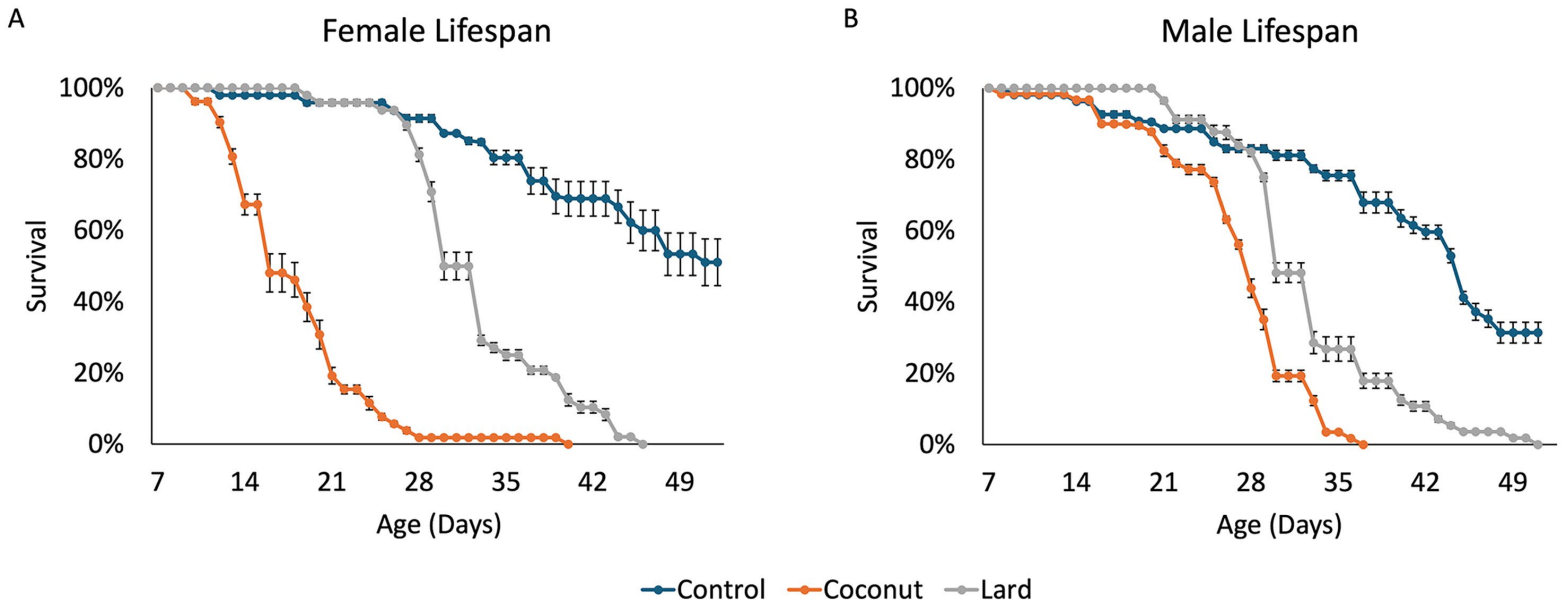
Effects of high-fat diets (HFD) containing 30% (w/v) of lard or coconut oil on the lifespan of male and female *Drosophila melanogaster*. **(A)** Both HFDs significantly reduced the lifespan of female *w^1118^* flies compared to controls (Log-rank *p* < 0.001). Furthermore, females fed coconut oil experienced significantly shorter lifespans than those fed lard (Log-rank *p* < 0.001, *n* = 51 control, 52 lard, 53 coconut oil). **(B)** Both HFD groups significantly reduced the lifespan of male *w^1118^* flies compared to controls. Both dietary oils also differed significantly from each other (Log-rank *p* < 0.001, *n* = 55 control, 60 coconut oil, 60 lard).

We first evaluated the influence of our dietary formulations for a HFD (30%, w/v) diet on the overall health of the fly by observing its effects on their lifespan. Two types of dietary fat, coconut oil and lard, were used to determine whether the ratio of saturated to unsaturated fat affected lifespan. *w^1118^* female flies experienced a significantly shorter mean and median lifespan on both dietary fat sources (Figure 1A and Table 1). Interestingly, the dietary source of fat significantly influenced this phenotype, with the females reared on coconut oil, higher in saturated fat, displaying a significantly lower mean lifespan of 18.1 ± 0.8 days compared to lard-reared females with 32.7 ± 0.9 days (*p* ≤ 0.001). Male *w^1118^* flies also mirrored the female phenotype with both oils reducing lifespan compared to controls, as well as significantly decreased for coconut oil with a mean lifespan of 27 ± 0.8 days compared to lard with 32.6 ± 0.9 days (Figure 1B and Table 1) (*p* ≤ 0.001).

**Table 1:** Mean and median lifespan for female and male *Drosophila* reared on either coconut, lard, or control diet.

Dietary treatment	Female		Male	
	Mean (Days, S.E.)	Median (Days, 95% C.I.)	Mean (Days, S.E.)	Median (Days, 95% C.I.)
Control	44.3 ± 1.4	45.0 ~ 51.0	40.9 ± 1.6	41.0 ~ 46.0
Coconut	18.1 ± 0.8	16.0 ~ 19.0	27 ± 0.8	27.0 ~ 28.0
Lard	32.7 ± 0.9	30.0 ~ 32.0	32.6 ± 0.9	30.0 ~ 32.0

### 3.2 Prolonged exposure to coconut oil in female flies results in behavioral deficits as well as neurodegeneration

**Figure 2:**
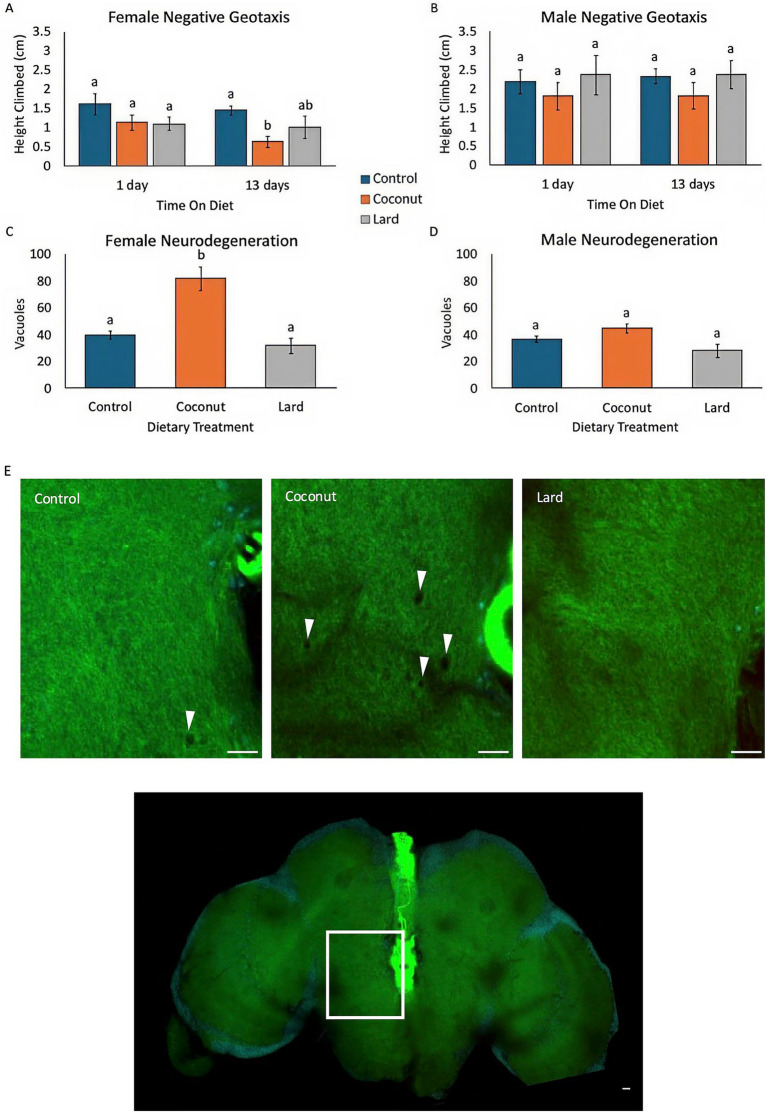
Behavioral and morphological effects of long-term exposure to high-fat diets (HFD) consisting of 30% (w/v) coconut oil or lard. **(A)** A reduction in climbing ability was observed in female flies fed coconut oil, but only after chronic exposure to the diet. There were no effects on climbing ability in females fed lard or in either male group (coconut oil or lard) at either time point **(B)**. *n* = 7 control, 8 coconut oil, 8 lard for 1-day females; 9 control, 10 coconut oil, 8 lard for 13-day females, 7 control, 8 coconut oil, 5 lard for 1-day males; 12 control, 11 coconut oil, 6 lard males for 13-day males. **(C,E)** Only females on the coconut oil diet showed an increase in neuronal vacuoles [white arrowheads in **(E)** compared to age-matched control and lard-fed flies (*n* = 9 control, 10 coconut oil, 6 lard)]. **(D)** No significant differences in vacuole count were found in males for any dietary fat (*n* = 6 control, 7 coconut oil, 5 lard). All data are presented as means ± SEM and analyzed using Two-Way ANOVA with Tukey’s HSD *post hoc* test. Scale bars represent 10 μm. The whole brain image is a composite of two 40X objective images, with insets showing single 60X objective taken from region outlined by white box.

Negative geotaxis refers to a well-characterized, instinctual predilection to climb in *Drosophila* (Gargano et al., 2005). This behavior is often used as a measure to study neuromuscular health and is known to decline with aging and amongst fly models of neurodegenerative disease. To further understand saturated fat’s influence on *Drosophila* neuronal health, we also wanted to investigate its effects under acute and chronic exposure. To do so, independent negative geotaxis assays were conducted after two lengths of exposure to the HFD: a single day and 13 days. As shown in Figure 2A, a single day of exposure to either diet did not affect climbing ability for mated females. When exposed to coconut oil, they were able to climb 1.12 ± 0.20 cm within 3 s of being forced to the bottom of the vial. This was not statistically different from the heights climbed by mated female flies on control (1.60 ± 0.28 cm) or lard (1.08 ± 0.17 cm) diets within the same time frame. After 13 days of exposure to the different dietary treatments, the coconut oil-fed group’s average climbing ability was 0.61 ± 0.15 cm, compared to age-matched control flies at 1.44 ± 0.12 cm (*p* ≤ 0.05). Lard-fed female flies’ climbing height was not statistically different from controls at 0.99 ± 0.29 cm. Additionally, no significant difference was seen at either time point in either dietary fat group for male flies compared to controls (Figure 2B).

We next investigated whether a HFD directly affects the *Drosophila* CNS by assessing brains for signs of tissue degeneration, as indicated by vacuoles. Following 13 days of dietary exposure, coconut oil–fed female flies exhibited a significant, approximately twofold increase in the number of vacuoles within the central brain lobes compared to age-matched controls (Figures 2C,E). This increase suggests that coconut oil consumption induces structural disruption or neuronal loss within this region. In contrast, females maintained on a lard-based diet did not display any differences in the amount of vacuolization (Figure 2C), indicating that the neurotoxic effects of dietary fat may depend on its source or composition. Furthermore, none of the male groups examined displayed detectable differences in brain morphology under either dietary condition (Figure 2D), suggesting that the CNS of female flies may be more susceptible to the detrimental effects of specific high-fat diets, as was observed with the climbing phenotype. A two-way ANOVA revealed main effects for both sex and diet as well as an interaction of the two.

### 3.3 High-fat diet’s effects on neuronal oxidative stress are time, sex, and dietary source specific

**Figure 3:**
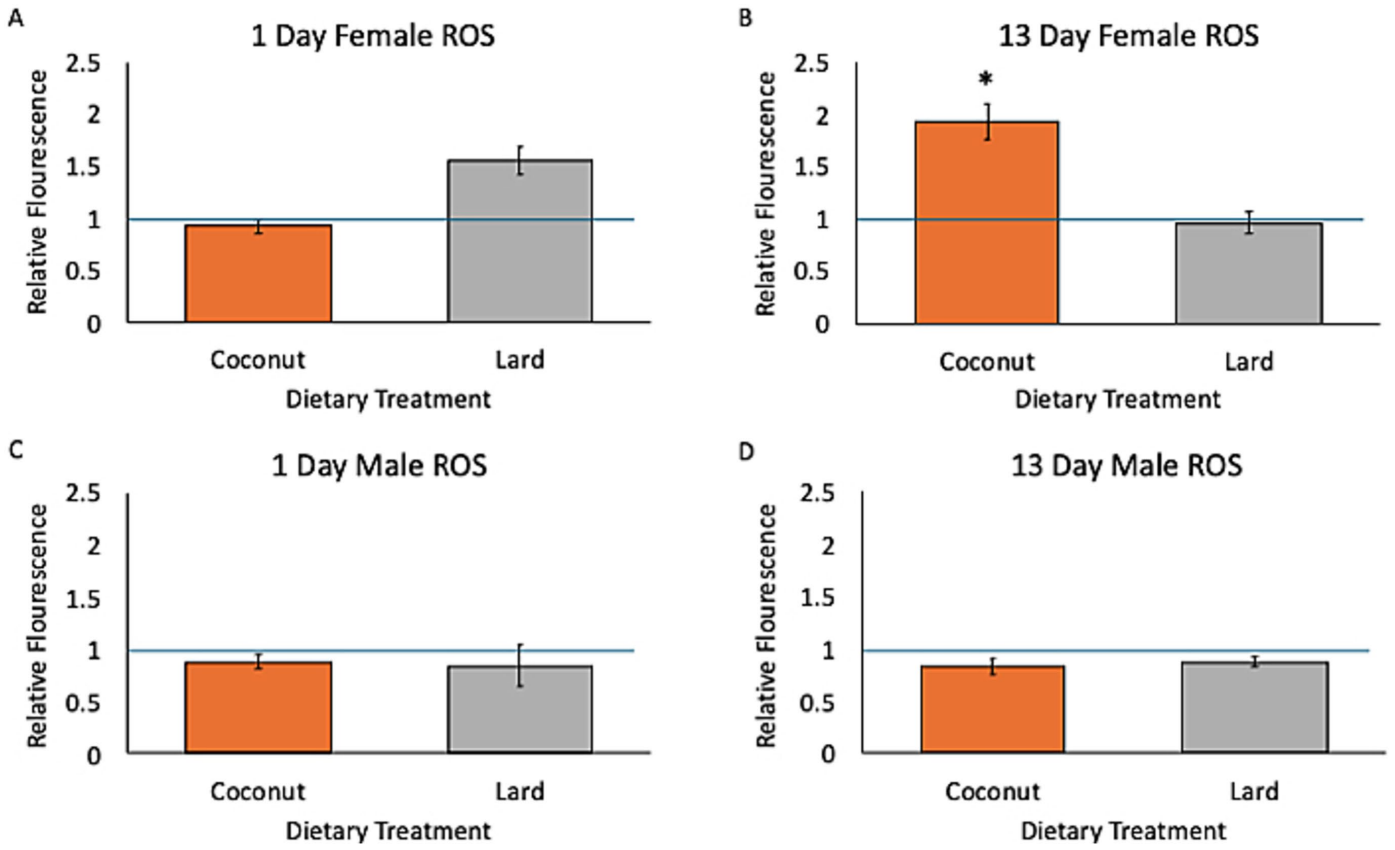
Differential effects of high-fat diet (HFD) on oxidative stress, depending on dietary exposure length, dietary oil type, and sex. **(A)** After 1 day of dietary treatment, female flies fed lard, but not coconut oil, showed a modest, though non-significant, increase in neuronal ROS compared to controls (blue line) (*n* = 7 control, 11 coconut oil, 5 lard). **(B)** After 13 days, females fed coconut oil, but not lard, exhibited a significant increase in neuronal ROS compared to controls (*n* = 15 control, 13 coconut oil, 8 lard). Male flies exposed to either a single day **(C)** or 13 days **(D)** of coconut oil or lard displayed a difference in neuronal ROS compared to their age-matched controls (*n* = 18 control, 21 coconut oil, 11 lard for 1-day; 21 control, 10 coconut oil, 8 lard for 13-day). All data are presented as means ± SEM, *p < 0.016, 2-tailed Student’s *T*-test with Bonferroni correction.

High levels of oxidative stress are known to be detrimental to neuronal homeostasis and have been shown to be involved in neurodegeneration. After a single day of exposure to the diet, female brains on the coconut oil diet displayed ROS levels comparable to control animals (Figure 3A), indicating an absence of oxidative stress in response to the diet. While ROS levels appeared elevated in the lard-fed group, this increase did not withstand Bonferroni correction. Chronic exposure to the diets resulted in differential states of oxidative stress between the two dietary fats. ROS levels were comparable to controls in lard-fed female flies after 13 days on the diet, whereas a two-fold increase in neuronal ROS levels was exhibited by the coconut oil-fed females (Figure 3B). Male flies experienced a different pattern of oxidative stress; neither the coconut oil nor the lard diet had a significant effect on neuronal ROS at either time point (Figures 3C,D).

### 3.4 Coconut oil does not influence NF-κB levels or activation in female flies

**Figure 4:**
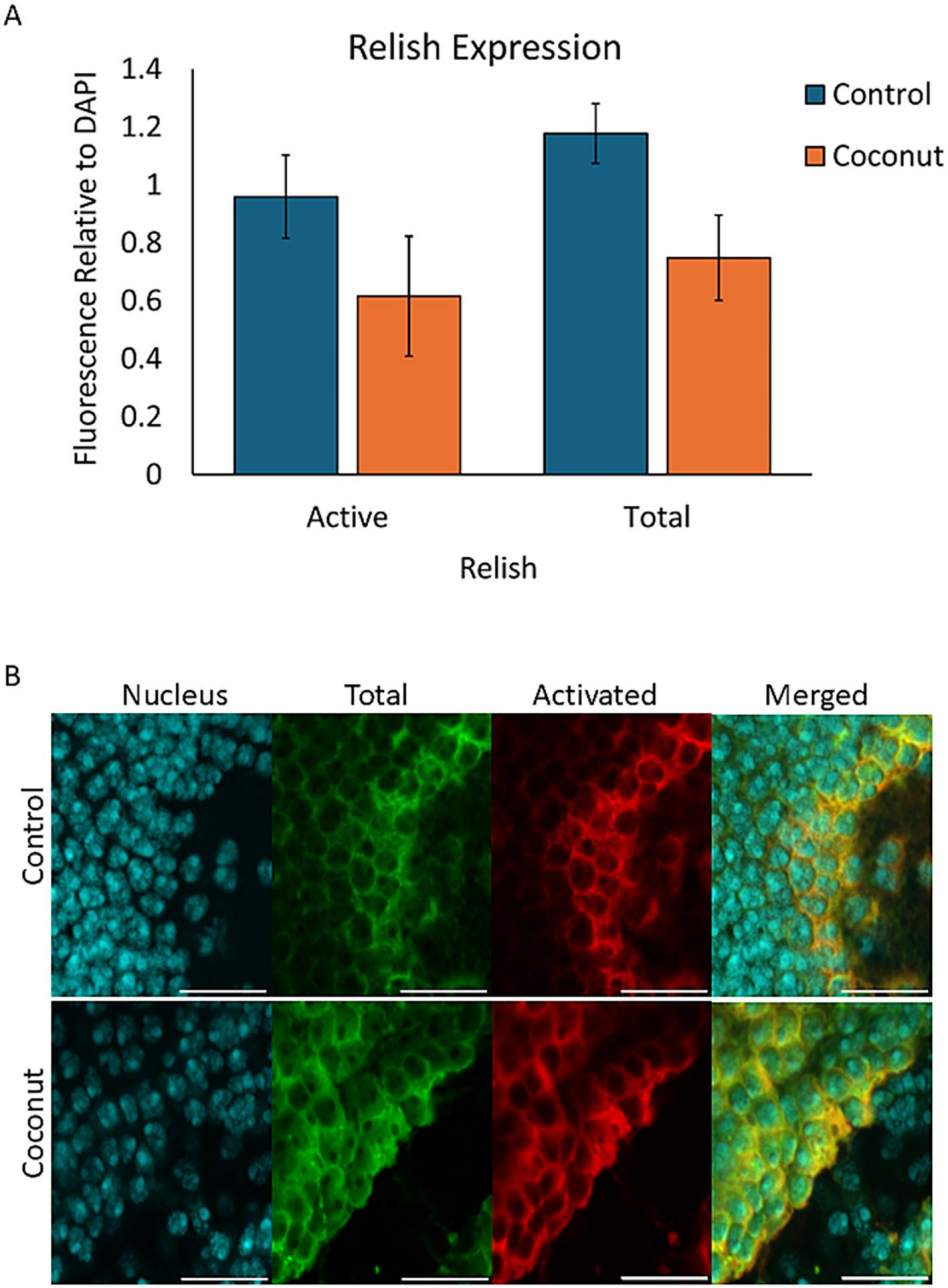
High-fat diet is not associated with an increase in *Drosophila* Relish expression or activation. **(A)** No significant differences were observed in the fluorescent antibodies for either the total Relish protein or the cleaved, activated form as measured by Fiji quantification. **(B)** Additionally, no translocation of the activated Relish (red) to the nucleus (blue, DAPI) was observed. All data are presented as means ± SEM and analyzed using a 2-tailed Student’s *T*-test, *n* = 6 control, 5 coconut oil. Images were taken on Nikon confocal using a 60x objective with 2.5x zoom, scale bars are 10 μm.

To investigate the possible role neuroinflammation might play in the neurodegeneration phenotype, IHC was utilized to observe alterations in either expression or translocation of the *Drosophila* transcription factor NF-κB homolog *relish*. IHC revealed no change in the amount of *Relish* protein in brains when quantifying relative fluorescence (Figure 4A). Fluorescent imaging once again revealed no difference in the amount of the activated form of the Relish protein, and representative images from both control and coconut oil HFD groups demonstrate that the protein does not overlap with the nucleus (Figure 4B).

### 3.5 Coconut oil high-fat diet slows autophagic flux, eventually resulting in an overall increase in autophagic vesicles

**Figure 5:**
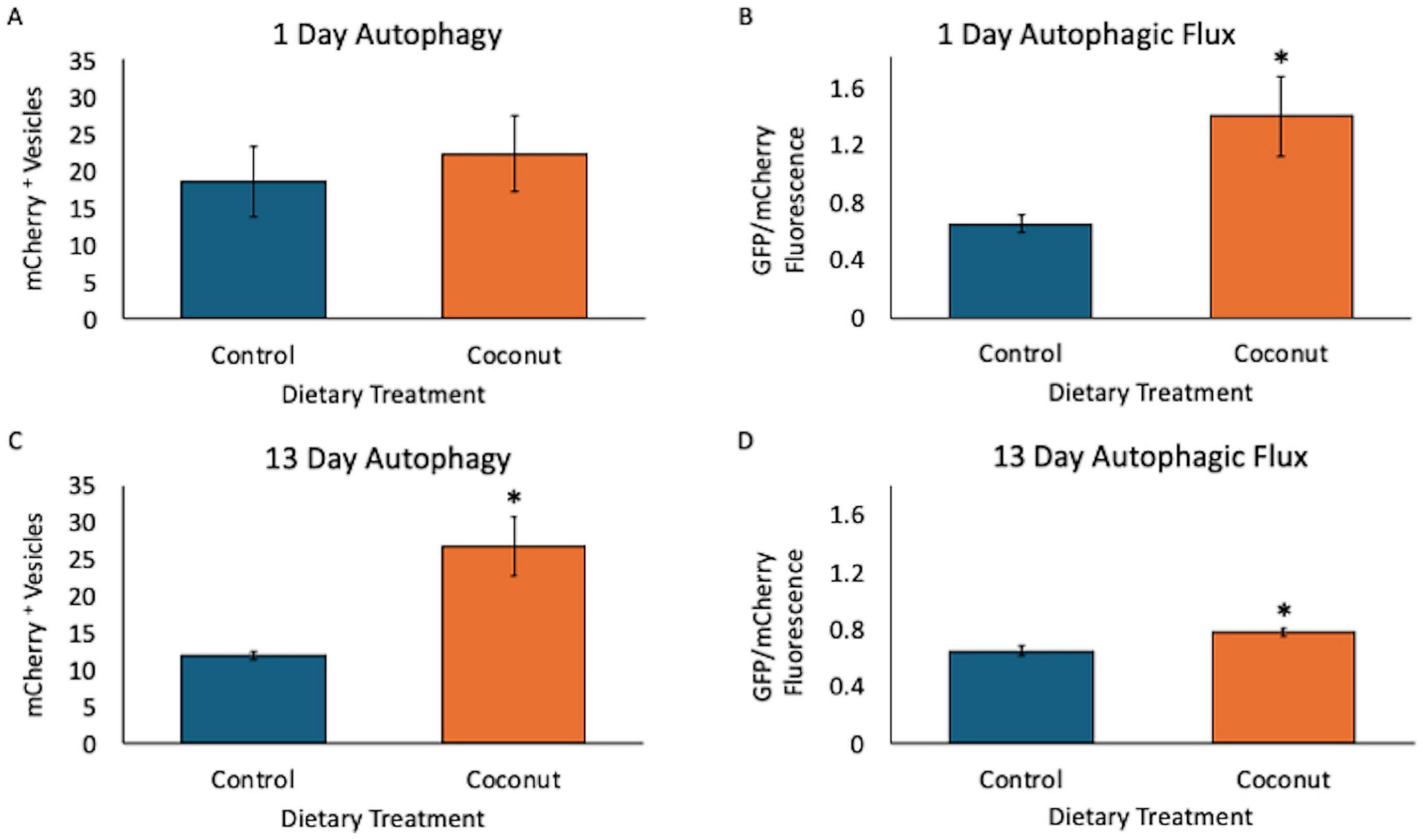
High-fat diet increases the number of autophagic vesicles and leads to a shift in autophagic flux that precedes the vesicle increase in females. **(A)** A single day on coconut oil HFD does not increase the number of autophagic vesicles, but chronic exposure does. **(B)** Ratios of the GFP/mCherry fluorescence reveal that more autophagosomes are present than autolysosomes, indicating a decrease in autophagic flux after only a single day on HFD. **(C)** Chronic exposure to coconut oil HFD increases the number of autophagic vesicles present in female fly brains. **(D)** GFP/mCherry ratios indicate that autophagic flux is also decreased after 13 days on HFD. These results suggest that the decrease in autophagic flux precedes changes to overall autophagy levels. All data are presented as means ± SEM and analyzed with 2-tailed Student’s *T* test. *n* = 6 control and 5 coconut oil for 1 day, *n* = 3 control and 4 coconut oil for 13 day. *p-value < 0.05.

Given coconut oil’s influence on neuronal oxidative stress levels in female flies, we next sought to observe its effects on autophagy within the primary cells of CNS lipid metabolism, glia, in female flies. This was accomplished using the glial specific gal-4 driven tandem GFP-mCherry ATG8a reporter line, which double tags autophagic vesicles with a green and red fluorescent marker. No changes to autophagy levels were evident after a single day on the coconut HFD, as exhibited by similar amounts of autophagic vesicles present within three different ROIs per sample (Figure 5A). While flies with chronic exposure to the diet displayed an increase in the number of autophagic vesicles present, with coconut oil-fed flies exhibiting an average of 26 vesicles per ROI and control-fed flies exhibiting 11 vesicles per ROI (Figure 5C).

Although no immediate changes in the number of autophagic vesicles were evident, the tandem reporter line allows visualization of autophagy speed, also known as autophagic flux, by comparing the two fluorescent signals. The pH-sensitive GFP signal is quenched in the acidic lysosomes involved in the degradation stage of autophagy, while the more stable mCherry signal remains detectable in both autophagosomes (the early, engulfment stage) and lysosomes. The ratio of GFP to mCherry fluorescence, therefore, provides a measure of autophagic flux. After a single day on the coconut oil HFD, a decrease in CNS autophagic flux was observed, as shown by the increase in GFP/mCherry ratio (Figure 5B). This suggests that although overall autophagy levels have not yet been affected by the coconut oil HFD, there is an immediate impact on the CNS autophagic flux. This decrease in flux persists even after 13 days on the HFD, although it is less severe (Figure 5D).

## 4 Discussion

Diet is one of the modifiable lifestyle factors that contributes to the risk of developing dementia and neurodegeneration (Livingston et al., 2020). A diet high in saturated fat has been shown to lead to detrimental health effects that may drive this increased risk, yet key questions remain regarding the underlying cellular processes driving neurodegeneration and how diet may influence them. This is especially difficult to study in humans and other mammalian models due to ethical constraints and the time and cost necessary to understand the influence of a lifelong dietary pattern on a disease typically manifesting in older adults. Because of this constraint, *Drosophila melanogaster* is uniquely positioned to serve as a model for the study of age-related neurodegenerative disease.

We demonstrated that a high-fat (30%, w/v) diet using dietary fats such as coconut oil or lard is sufficient to decrease the mean lifespan of flies. Others have observed this phenotype with various dietary fat formulations, demonstrating that a concentration as low as 3% is enough to significantly reduce lifespan when butterfat is the dietary source (Eickelberg et al., 2022). Other studies have utilized different dietary fats, including coconut oil and lard, typically at concentrations between 10 and 30% (w/v) and reliably reproduce a shortened lifespan (Liao et al., 2021; Rivera et al., 2019). Few studies have directly compared multiple fat sources within the same experiment, and to our knowledge, none have compared coconut oil versus lard in this context, despite both being widely used in *Drosophila* studies. One study conducted by Eickelberg et al. (2022) compared several dietary oils and found that while each had unique effects on lifespan and climbing ability, the group with the highest saturated-fat content (butterfat) produced a smaller decrease in lifespan than sunflower oil, olive oil, linseed oil, or fish oil in female flies, and did not impair climbing. While this study did not include coconut oil or lard, its diet contained only 12% fat, and butterfat has a lower saturated-fat content than coconut oil (63% vs. 92%) and is similar to lard (63% vs. 42%). Thus, the lack of climbing impairment in their butterfat group aligns with our lard-fed females, highlighting that the markedly higher saturated-fat content of coconut oil in the context of a higher total-fat diet likely drives the more severe behavioral decline observed here. It should be noted that visual exposure to dead flies has been shown to influence lifespan and the brain metabolome (Chakraborty et al., 2019). Dead flies were removed when food was refreshed (every 2–3 days) for our experiments requiring aging of the animals, so dietary and control groups likely had visual exposure to dead animals at some point during their aging. The extent of this influence on the HFD-related phenotypes warrants further investigation.

Few papers have examined the direct effects of HFD within the *Drosophila* CNS. Among these, evidence supports sex-specific alterations to neuronal function and gene expression in response to HFD (Liao et al., 2021; Stobdan et al., 2019; Huang et al., 2020; Hemphill et al., 2017; Nayak and Mishra, 2021). For example, Stobdan et al., reported that female heads exhibited differential expression among genes involved in fatty acid metabolism, particularly an up-regulation of mitochondrial ß-oxidation genes after exposure to HFD, whereas males showed reduced expression of stress-response genes (Stobdan et al., 2019). Similarly, Rivera et al. found that heads of HFD-fed females displayed differential expression of genes involved in oxidative stress, immune response, feeding behavior, and memory, demonstrating impaired memory retention after HFD exposure (Rivera et al., 2019). While these studies reported oxidative stress-associated transcriptional changes, none detected changes in genes associated with autophagy, potentially due to differences in diet composition, duration, or tissue specificity (whole heads versus isolated brains). Whole-head sequencing may obscure CNS-specific responses due to contributions from the eye, cuticle, and fat-body tissue. Liao et al. also observed dopaminergic neuron deficits in females after 3 weeks on a coconut oil HFD. They reported a decrease in the presence of the dopamine synthesis rate-limiting enzyme, tyrosine hydroxylase, indicating a reduction in dopamine-synthesizing neurons in response to the HFD (Liao et al., 2021). To our knowledge, our work is the first to show direct structural neurodegeneration as evidenced by an increase in vacuolization of the brains of HFD-fed female flies. Taken together, our results add to the body of evidence supporting the detrimental effects of a high saturated fat diet on the functionality and integrity of the fly’s CNS. Two-way ANOVA revealed a diet-by-sex interaction, indicating a female-specific vulnerability to saturated fat, consistent with prior studies and known sex differences in fly lipid metabolism (Liao et al., 2021; Stobdan et al., 2019; Eickelberg et al., 2022; Rivera et al., 2019).

Neuroinflammation is a hallmark of many neurodegenerative diseases, often marked by altered cytokine profiles such as TNF-α and IL-6 (Duan et al., 2018). Accordingly, we investigated activation of Relish, the *Drosophila* NF-κB homolog. Surprisingly, no change was observed in either total or activated Relish in HFD-fed females. Although the NF-κB signaling pathway plays a primary role in propagating neuroinflammation, its involvement has mixed support. Indeed, most studies investigating HFD’s effects on neuroinflammation look at indirect effects of NF-κB, such as upstream and downstream targets, rather than activation of NF-κB itself. One 2018 study found an increase in the cerebral expression of NF-κB in male mice fed an HFD for 16 weeks, but it should be noted that this study utilized soybean oil as their dietary fat source which may, in part, explain the differences observed in our results (Nuzzo et al., 2018). Additionally, they looked at overall expression of the protein via western blotting, therefore levels of activation or translocation of the protein to the nucleus remain unknown. Another study found that mice fed an HFD for 8 weeks experienced a downregulation in hypothalamic NF-κB and TNF-α, yet 20 weeks of the diet showed a significant increase in TNF-α. NF-κB was not measured at this time point. Further investigation into the more direct effect saturated fat has on these neurons revealed that immortalized hypothalamic cell culture lines exposed to palmitate increased the expression of TNF-α but not of NF-κB (Dalvi et al., 2017). It should also be noted that these studies were done in males, which may contribute to the discrepancy between our findings and previously published data. One of the few studies to investigate male and female mice found that only males showed an increase in proinflammatory cytokines IL-1ß, IL-6, and TNF-α in the hypothalamus; females showed no changes to any markers of neuroinflammation (Morselli et al., 2014). Another study found that markers of neuroinflammation were only elevated in female mice on a 60% HFD but not 45% HFD, whereas males had elevated markers in both (Murtaj et al., 2022). Our findings therefore align with a sexually dimorphic neuroinflammatory response and suggest that saturated fat-induced neurodegeneration in flies may proceed independently of Relish (NF-κB) activation, highlighting the need to examine alternative inflammatory signaling pathways like the Toll/IMD, JAK/STAT pathway, and antimicrobial peptides.

Autophagy plays a pivotal role in neuronal homeostasis, nutritional adaptation, and oxidative-stress mitigation. Given this, its disruption has emerged as a key driver of neurodegeneration. As a multi-step pathway that is heavily regulated at each stage, the rate at which autophagy progresses from initiation to degradation matters. For example, an increase in only the earlier stages may make it appear that autophagy is increasing, but because the material is not degraded, it is detrimental rather than beneficial to the cell. We observed decreased autophagic flux in glia, the primary CNS lipid-processing cells, manifested by accumulation of early-stage autophagosomes. This phenotype parallels mammalian HFD studies showing increased autophagy-associated proteins and changes in the ratio between LC3 1 and LC3 2, which differentiate between autophagosomes and autolysosomes (Yi et al., 2024; Hou et al., 2022; Li et al., 2022; Wu et al., 2024). As in our study, these results indicate that there is a higher formation of autophagosomes than degradation of autolysosomes, or a decrease in autophagic flux. Because ATG8a, the fly homolog of LC3, also accumulated, our findings support a conserved autophagy impairment mechanism across species (Demir and Kacew, 2023). Since autophagy also degrades ROS and damaged mitochondria (Santarelli et al., 2023), impaired flux may compound oxidative stress over time, aligning with our observed delayed ROS elevation.

In conclusion, this study demonstrates the value of *Drosophila melanogaster* as a nutritional model for diet-induced neurodegeneration. We show that high saturated fat intake decreases lifespan and climbing ability and uniquely induces structural neurodegeneration in females. While coconut oil HFD did not acutely elevate ROS, aging flies displayed increased oxidative stress, underscoring that lifelong saturated fat exposure, not short-term diet, is detrimental to neuronal integrity. Furthermore, while NF-κB homolog, *relish*, signaling did not appear to mediate coconut oil-induced neurodegeneration, impaired autophagic flux emerged as a key mechanism, consistent with mammalian findings. Together, these results highlight *Drosophila* as a cost-effective, genetically tractable system for dissecting diet-brain interactions and suggest that saturated fat-driven neurodegeneration may be mediated more by metabolic stress and autophagy disruption than classical inflammatory pathways. Future work using the powerful genetic tools available in flies will enable precise mechanistic dissection of sex-specific vulnerability and metabolic-stress pathways in diet-induced brain aging.

## Data Availability

The raw data supporting the conclusions of this article will be made available by the authors, without undue reservation.
